# Influence of reconsolidation in maintenance of cocaine-associated contextual memories formed during adolescence or adulthood

**DOI:** 10.1038/s41598-023-39949-y

**Published:** 2023-08-25

**Authors:** André N. Herrera Charpentier, Doris I. Olekanma, Christian T. Valade, Christopher A. Reeves, Bo Ram Cho, Amy A. Arguello

**Affiliations:** Department of Psychology, Behavioral Neuroscience, Michigan State University (MSU), Interdisciplinary Science and Technology Building, West Rm. 4010, 766 Service Rd., East Lansing, MI 48824 USA

**Keywords:** Addiction, Operant learning

## Abstract

Adolescents are at increased risk to develop substance use disorders and suffer from relapse throughout life. Targeted weakening of drug-associated memories has been shown to reduce relapse-like behavior in adult rats, however this process has been understudied in adolescents. We aimed to examine whether adolescent-formed, cocaine-associated memories could be manipulated via reconsolidation mechanisms. To accomplish this objective, we used an abbreviated operant cocaine self-administration paradigm (ABRV Coc-SA). Adult and adolescent rats received jugular catheterization surgery followed by ABRV Coc-SA in a distinct context for 2 h, 2×/day over 5 days. Extinction training (EXT) occurred in a second context for 2 h, 2×/day over 4 days. To retrieve cocaine-context memories, rats were exposed to the cocaine-paired context for 15 min, followed by subcutaneous injection of vehicle or the protein synthesis inhibitor cycloheximide (2.5 mg/kg). Two additional EXT sessions were conducted before a 2 h reinstatement test in the cocaine-paired context to assess cocaine-seeking behavior. We find that both adult and adolescent cocaine-exposed rats show similar levels of cocaine-seeking behavior regardless of post-reactivation treatment. Our results suggest that systemic treatment with the protein synthesis inhibitor cycloheximide does not impair reconsolidation of cocaine-context memories and subsequent relapse during adulthood or adolescence.

## Introduction

Substance-use disorders (SUDs) are a chronic disease characterized by cycles of repeated use, abstinence, and relapse. Patients that suffer from SUDs often report starting drug use during adolescence^1,2^, with recent reports showing that the severity of SUD diagnosis during adolescence increases the risk for problematic drug use in adulthood^3,4^. Cocaine use disorders affect up to 1.5 million people in the US, with reports of increased cocaine use in adolescents and young adults from 2005 to 2018, highlighting the importance of studying relapse during this developmental period^5,6^.

The cues and contextual environments linked with drug use are major triggers for relapse^7–9^. Exposure to cocaine-associated contexts can elicit craving and relapse in part through the retrieval of cocaine-associated memories^10–12^. Normally, when memories are retrieved, they enter a temporary labile state and can be reconsolidated back into long-term storage^13–15^. Each drug-taking or relapse episode can serve to strengthen cocaine-associated memories. Targeted weakening of maladaptive drug-related memories, via reconsolidation mechanisms, has been proposed as a potential therapeutic method to reduce the risk to relapse^16–18^. The process of reconsolidation is dependent on new protein synthesis, with reduced cocaine conditioned place preference (CPP), cue- and context-elicited cocaine seeking observed in adult rats when protein synthesis is inhibited during the labile window^10,19–21^.

There is evidence that adolescent fear-associated and social CPP memories are sensitive to reconsolidation-based manipulations^22–25^. However, much less is known about the potential lability of drug-associated memories formed during adolescence, despite the critical role of this developmental period in initiation of drug use^18^. We therefore examined whether protein synthesis inhibition would impair the reconsolidation of adult or adolescent-formed cocaine-context-memories and subsequent relapse-like behavior. We used a published, abbreviated cocaine self-administration model (ABRV Coc-SA) that allows for self-administration training and reinstatement testing in rats to occur during the adolescent window, between postnatal days 35–63^26,27^. We modified the ABRV Coc-SA paradigm by incorporating a memory reactivation session to elicit retrieval of cocaine-associated contextual memories in adult and adolescent rats, followed by systemic protein synthesis inhibition before a test of cocaine-seeking behavior.

## Materials and methods

### Animals

For all experiments, male Sprague Dawley rats (Envigo Inc., Haslett, MI) arrived on postnatal day 25 (P25, average of 75 g, n = 14), or P56 (average of 257 g, n = 16). All rats were pair housed upon arrival with ad libitum access to food and water. The vivarium housing was humidity and temperature controlled under reversed light conditions (8 am off, 8 pm on) with all behavioral experiments conducted during the rats’ active cycle. Rats were handled 6–7 days with jugular catheterization surgeries performed on P33 for adolescent or P66 for adult rats.

Previous reports did not find behavioral differences associated with single- vs pair-housed conditions using ABRV Coc-SA, therefore after a surgery and recovery period (2 days before behavioral start) all rats were single housed with adolescents maintained on 15 g and adults on 18 g of chow, which allowed for weight gain throughout the experiment^27,28^. All protocols were approved by the Institutional Animal Care and Use Committee (IACUC) at Michigan State University (MSU) and followed the National Research Council’s Guide for the Care and Use of Laboratory Rats.

### Surgery

Intravenous (IV) catheters were constructed in house, with a 10 cm length for adults and 9.7 cm length for adolescents. A silicone ball was placed 3.25 or 3.0 cm from the inserted end of the catheter for adults or adolescents^27–29^. On the surgery day, rats were fully anesthetized via intraperitoneal (IP) injection of ketamine + xylazine (80–100 mg/kg + 5–10 mg/kg, respectively; Covetrus, Cats:11695-6840, 33198). Catheters were implanted into the right jugular vein, exiting subcutaneously at the back below the shoulder blade^27,30,31^. Rats were administered oral meloxidyl 2 days pre- and post surgery (Covetrus, Cat:50401), topical lidocaine and gentamycin applied to suture areas, and catheters flushed with 0.1 mL of cefazolin (0.1 mg/mL, Covetrus, Cat:54846) dissolved in heparinized saline (Hep-Sal; 70 U/mL; Covetrus, Cat:49130), followed by 0.1 mL of 10 U/mL Hep-Sal. Catheters were flushed before Coc-SA with 70U Hep-Sal, and after with 10U Hep-Sal. Cefazolin was administered after the last Coc-SA session of the day. When catheter patency was uncertain, 0.1 mL propofol was administered IV and rats monitored for brief loss of movement (Covetrus, Cat:54899).

### General Coc-SA training

**Figure 1:**
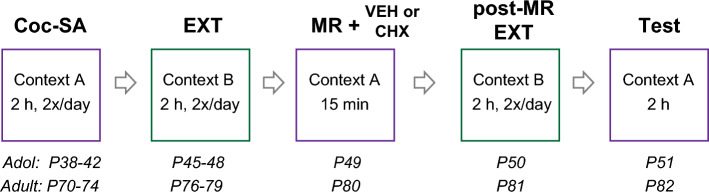
Schematic and timeline of behavioral experiments to examine reconsolidation of cocaine-context memories in adult and adolescent cocaine-exposed rats. All experiments included abbreviated cocaine self-administration training (Coc-SA, 2 h sessions, 2×/day over 5 days, minimum of 10 sessions) in a distinct context, followed by extinction training (EXT, 2 h sessions, 2×/day over 4 days, minimum of 8 sessions). On average, adolescent rats started Coc-SA at P38 and adults at P70. The average age at the start of EXT was P45 for adolescent rats and P76 for adult rats, due to differences in the number of sessions needed to acquire Coc-SA criteria. To retrieve cocaine-associated contextual memories, rats were returned to the previous cocaine-paired context for memory reactivation (MR, 15 min session), followed by a subcutaneous injection of vehicle or 2.5 mg/kg cycloheximide (VEH, CHX) immediately after the MR session. Two additional sessions of EXT were given 1 day after MR (post-MR EXT), followed by a reinstatement test in the previous cocaine-paired context (Test, 2 h).

As shown in Fig. 1, Coc-SA occurred in an operant conditioning chamber with distinct odor, tactile, auditory and visual stimuli, as previously described^27^. Operant chambers (29.5 × 24 × 28 cm; Med Associates Inc., St. Albans, NY) were configured with the following contextual stimuli: Context A contained a continuous red house light (0.4 fc brightness), intermittent pure tone (80 dB, 1 kHz; 2 s on, 2 s off), pine-scented air freshener (Car Freshener Corp., Watertown, NY), and wire mesh flooring (26 cm × 27 cm). Context B contained an intermittent white stimulus light over the inactive lever (1.2 fc brightness; 2 s on, 2 s off), continuous pure tone (75 dB, 2.5 kHz), vanilla-scented air freshener (Car Freshener Corp., Watertown, NY), and a slanted black acrylic panel bisecting the bar flooring (19 cm × 27 cm). Rats were counter-balanced to start Coc-SA in context A or B and importantly, background stimuli were not paired with cocaine infusion^8,27,32^.

Coc-SA consisted of 2 h sessions, 2×/day over 5 days, for a minimum of 10 sessions (criteria = at least 10 infusions). Active lever responses resulted in an IV infusion of 0.05 mL of Cocaine-HCl (0.5 mg/kg per each infusion) on a Fixed-Ratio 1 (FR1) schedule of reinforcement, whereas inactive lever responses resulted in no infusions. Cocaine hydrochloride (Cocaine-HCl; NIDA Drug Supply System, Research Triangle Park, NC) was dissolved in sterile saline. Drug delivery was controlled by an infusion pump (Med Associates Inc; Model PHM-107) and each infusion lasted for 2 s with a 20 s time-out period. Weight was recorded daily and cocaine concentration in syringes adjusted for every 50 g increase in weight^27,33^. As shown in Fig. 1, Coc-SA began on P38 (adolescent) or P70 (adult), and importantly at a post-pubertal period for adolescents^26^. Rats were returned to their home-cage environments for a break period of 2 h between training sessions.

### Extinction training, memory reactivation and reinstatement tests

As shown in Fig. 1, extinction training (EXT) consisted of 2 h sessions, 2×/day over 4 days, for a minimum of 8 sessions (criteria = less than 25 responses on last 2 sessions). During EXT training, responses on the active and inactive lever had no programmed consequences. Rats received a 15 min memory reactivation session (MR) in the cocaine-paired context. The MR session functions to elicit brief memory reactivation, without extensive extinction^11,34^. Immediately after MR, rats were given a subcutaneous (SC) injection of vehicle (VEH) or cycloheximide (CHX, 2.5 mg/kg) and returned to their home cage. We used a 2.5 mg/kg dose based on previous studies which showed that 2.2 mg/kg given immediately after retrieval of a cocaine-cue memories diminished subsequent relapse^21^. To assess whether CHX affected responding in the EXT context, two additional 2 h EXT sessions were administered (post-MR EXT). After the final post-MR EXT, rats were returned to the original cocaine-paired context for a 2 h reinstatement test (Test). During the Test, both active and inactive lever presses resulted in no programmed consequences; therefore, active lever responses served as an index of drug-seeking behavior. On average, MR and Tests occurred on P49 and P51 for adolescent rats and P80 and P82 for adult rats.

### Statistical analysis

Separate analyses of variance (ANOVAs) or independent t-tests were conducted to examine for pre-existing differences between VEH and CHX groups for: lever responses during Coc-SA (Mean ± SEM, final 3 sessions), last EXT session, 15 min MR session, final post-MR EXT session, and number of sessions to reach acquisition criteria for Coc-SA and EXT.

**Figure 2:**
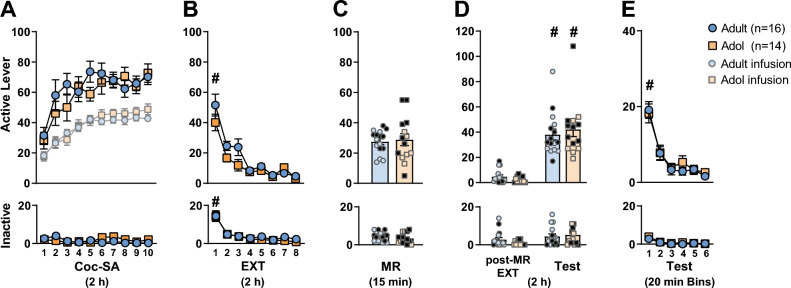
Effect of age on behavioral phases. Mean ± SEM of active and inactive lever responses for adult and adolescent rats during **(A)** cocaine self-administration (Coc-SA, 2 h sessions, 2×/day over 5 days), **(B)** extinction (EXT, 2 h sessions, 2×/day over 4 days), **(C)** memory reactivation (MR, 15 min session) in the previous cocaine-paired context to retrieve cocaine-associated memories. Rats received subcutaneous injection of vehicle or 2.5 mg/kg cycloheximide (VEH, CHX) immediately after the MR session. **(D)** Two additional sessions of EXT were given 1 day after MR (post-MR EXT), followed by a reinstatement test in the previous cocaine-paired context (Test, 2 h). **(E)** Active and inactive lever responses during 20 min Bins of the 2 h Test. Symbols indicate significant within-subject differences revealed by Tukey’s test **(B)** ^#^p < 0.01: EXT session 1 > 8, **(D)** final post-MR EXT session < Test, **(E)** 20 min Test Bin 1 > 6. Groups denoted by: Blue = adult (n = 16), Orange = adolescent (n = 14), Light blue and Light orange individual points = VEH, Black individual points = CHX.

To assess behavioral differences by age, data from adult and adolescent VEH and CHX groups pre-treatment were collapsed. Interaction effects for within subject factors. *Coc-SA, EXT, MR* and the between subject factor of *Age* were assessed (Fig. 2). For all behavioral phases (Coc-SA, EXT, MR, final post-MR EXT and Test), no *Age* × *Context* × *Treatment* interaction effects were found and therefore subsequent ANOVAs were analyzed separately for adult or adolescent groups.

**Figure 3:**
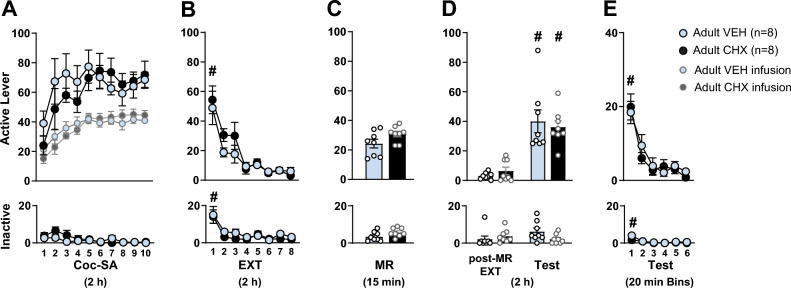
Effect of cycloheximide on reconsolidation of cocaine-context memories in adult cocaine-exposed rats. Mean ± SEM of active and inactive lever responses for adult rats during **(A)** cocaine self-administration (Coc-SA, 2 h sessions, 2×/day over 5 days), **(B)** extinction (EXT, 2 h sessions, 2×/day over 4 days), **(C)** memory reactivation (MR, 15 min session) in the previous cocaine-paired context to retrieve cocaine-associated memories. Adult rats received subcutaneous injection of vehicle or 2.5 mg/kg cycloheximide (VEH, CHX) immediately after the MR session. **(D)** Two additional sessions of EXT were given 1 day after MR (post-MR EXT), followed by a reinstatement test in the previous cocaine-paired context (Test, 2 h). **(E)** Active and inactive lever responses during 20 min Bins of the 2 h Test. Symbols indicate significant within-subject differences revealed by Tukey’s test **(B)** ^#^p < 0.01: EXT session 1 > 8, **(D)** final post-MR EXT session < Test, **(E)** 20 min Test Bin 1 > 6. Groups denoted by: Light blue = adult VEH (n = 8), Black = adult CHX (n = 8).

**Figure 4:**
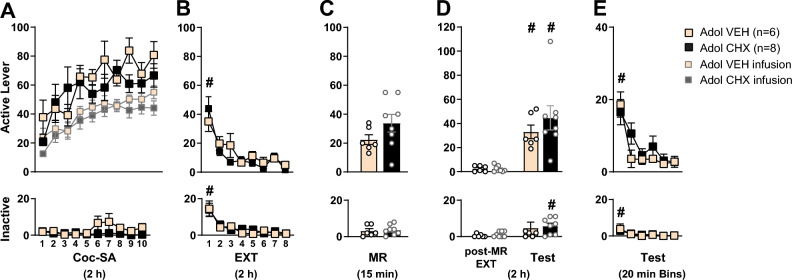
Effect of cycloheximide on reconsolidation of cocaine-context memories in adolescent cocaine-exposed rats. Mean ± SEM of active and inactive lever responses for adolescent rats during **(A)** cocaine self-administration (Coc-SA, 2 h sessions, 2×/day over 5 days), **(B)** extinction (EXT, 2 h sessions, 2×/day over 4 days), **(C)** memory reactivation (MR, 15 min session) in the previous cocaine-paired context to retrieve cocaine-associated memories. Adolescent rats received subcutaneous injection of vehicle or 2.5 mg/kg cycloheximide (VEH, CHX) immediately after the MR session. **(D)** Two additional sessions of EXT were given 1 day after MR (post-MR EXT), followed by a reinstatement test in the previous cocaine-paired context (Test, 2 h). **(E)** Active and inactive lever responses during 20 min Bins of the 2 h Test. Symbols indicate significant within-subject differences revealed by Tukey’s test **(B)** ^#^p < 0.01: EXT session 1 > 8**, (D)** ^#^p < 0.01, active; ^#^p < 0.05, inactive: final post-MR EXT session < Test **(E)** ^#^p < 0.01, active; ^#^p < 0.05, inactive: 20 min Test Bin 1 > 6. Groups denoted by: Light orange = adolescent VEH (n = 6), Black = adolescent CHX (n = 8).

To assess behavioral differences for adults (Fig. 3) or adolescents (Fig. 4), interaction effects for within-subjects factors: *Coc-SA, EXT, MR, Context* (final post-MR EXT session vs Test), 20 min Test Bins, 5 min MR or 5 min Test Bins and between subjects factor of *Treatment* (VEH vs CHX) were conducted. Significant effects, when appropriate, were followed by analysis with Tukey’s HSD post-hoc tests, with alpha set at 0.05. Rats that did not acquire Coc-SA or did not pass catheter patency tests were excluded from analysis.

### ARRIVE guidelines

The current study is reported in accordance with ARRIVE guidelines.

## Results

### Adolescent and adult behavior during Coc-SA, EXT, MR, and tests

To examine potential behavioral differences between adult and adolescent rats, lever response data for all phases before VEH or CHX administration (Coc-SA, EXT, MR) were combined by age. Overall, there were no pre-existing differences between adult and adolescent rats in the number of sessions to acquire Coc-SA, lever responses, or cocaine infusions during the last three sessions of Coc-SA (Fig. 2A). The 2 × 10 ANOVAs during Coc-SA did not reveal significant *Coc-SA session* × *Age* or *Coc-SA infusion* × *Age* interaction effects.

During EXT, there were no pre-existing differences between adult and adolescent rats in the number of sessions needed to complete EXT training, or lever responses on the last EXT session (Fig. 2B). The 2 × 8 ANOVA of lever responses revealed a significant *EXT session* main effect (active: F_7, 28_ = 44.395, p < 0.001; inactive: F_7, 28_ = 28.558, p < 0.001), but no *EXT session* × *Age* interaction or *Age* main effects. Tukey’s post-hoc comparisons revealed that both adult and adolescent groups decreased lever responses by the final EXT session (active and inactive: EXT S1 > S8, ^#^p < 0.01).

Adult and adolescent rats had similar lever responses during the 15 min MR session before systemic VEH or CHX administration (Fig. 2C). The 2 × 3 ANOVA of lever responses during 5 min Bins of the 15 min MR session revealed a significant *MR Bin* main effect (active: F_2, 28_ = 22.226, p < 0.001; inactive: F_2, 28_ = 3.646, p = 0.032) with no *MR Bin* × *Age* interaction or *Age* main effects. Tukey’s post-hoc comparisons revealed that both adult and adolescent groups displayed reduced responses on the active lever by the final 5 min of MR (active: Bin 1 > 3, ^#^p < 0.05) (Suppl Fig. 2A). Tukey’s post-hoc comparisons did not reveal significant differences in inactive lever responses during 5 min MR Bins (Suppl Fig. 2A).

To examine for potential behavioral differences between adult and adolescent rats during the reinstatement Test after VEH or CHX administration, we compared lever response data for (1) VEH groups only or (2) VEH and CHX groups combined by age and observed similar results. Therefore we presented data for all groups. Adult and adolescent groups displayed increased cocaine-seeking behavior when exposed to the cocaine-paired context (Fig. 2D). The 2 × 2 ANOVAs of active lever responses during the final post-MR EXT vs Test session revealed a significant *Context* main (active: F_1, 14_ = 56.826, p < 0.001), but no *Context* × *Treatment* interaction or *Treatment* main effects. Tukey’s post-hoc comparisons revealed that lever responses in the VEH and CHX groups were higher during the Test compared to the final post-MR EXT session (active: ^#^p < 0.01) therefore, both VEH and CHX-treated groups exhibited cocaine-seeking behavior. The 2 × 2 ANOVAs of inactive lever responses during the final post-MR EXT vs Test session did not reveal significant *Context* × *Treatment* interaction, *Context* or *Treatment* main effects.

The 2 × 6 ANOVA of lever responses during 20 min Test Bins of the 2 h Test revealed significant *Test Bin* main (active: F_5, 28_ = 42.136, p < 0.001; inactive: F_5, 28_ = 13.150, p < 0.001), with no *Test Bin* × *Age* interaction or *Age* main effects (Fig. 2E). Tukey’s post-hoc comparisons revealed that both adult and adolescent groups decreased active lever responses by the final 20 min of the Test (active: Bin 1 > 6, ^#^p < 0.01). Tukey’s post-hoc comparisons did not reveal significant differences in inactive lever responses during 20 min Test Bins.

For all behavioral phases, no *Context* × *Age* × *Treatment* interaction effects were found, therefore adult and adolescent groups were further independently analyzed.

### Adult behavior during Coc-SA, EXT, MR and tests: effect of CHX on reconsolidation of cocaine context memories

Adult rats in the VEH (n = 8) and CHX (n = 8) groups displayed similar responses on both levers during Coc-SA, with no pre-existing differences observed in the number of sessions to acquire Coc-SA, lever responses or cocaine infusions during the last three sessions of Coc-SA (Fig. 3A). The 2 × 10 ANOVAs during Coc-SA did not reveal significant *Coc-SA session* × *Treatment* or *Coc-SA infusion* × *Treatment* interaction effects.

Adult rats in the VEH and CHX groups had similar lever responses during EXT training (Fig. 3B). The 2 × 8 ANOVAs of lever responses revealed a significant *EXT session* main effect (active: F_7, 14_ = 26.439, p < 0.001; inactive: F_7, 14_ = 13.301, p < 0.001), with no *EXT session* × *Treatment* interaction or *Treatment* main effects. Tukey’s post-hoc comparisons revealed that both groups decreased lever responses by the final EXT session (active and inactive: EXT S1 > S8, ^#^p < 0.01).

Adult rats in the VEH and CHX groups had similar lever responses during the MR session (Fig. 3C). The 2 × 3 ANOVA of lever responses during 5 min bins of the 15 min MR session revealed a significant *MR Bin* main effect (active: F_2, 14_ = 14.646, p < 0.001) with no *MR Bin* × *Treatment* interaction or *Treatment* main effects. Tukey’s post-hoc comparisons revealed that both VEH and CHX groups displayed reduced active lever responses by the final 5 min of MR (active: Bin 1 > 3, ^#^p < 0.01). Tukey’s post-hoc comparisons did not reveal significant differences in inactive lever responses during 5 min MR Bins (Suppl Fig. 3A).

Adult rats in VEH and CHX groups displayed increased cocaine-seeking behavior when exposed to the cocaine-paired context (Fig. 3D). The 2 × 2 ANOVAs of active lever responses during the final post-MR EXT vs Test session revealed a significant *Context* main (active: F_1, 14_ = 56.826, p < 0.001), but no *Context* × *Treatment* interaction or *Treatment* main effects. Tukey’s post-hoc comparisons revealed that lever responses in the VEH and CHX groups were higher during the Test compared to the final post-MR EXT session (active: ^#^p < 0.01) therefore, both VEH and CHX-treated groups exhibited cocaine-seeking behavior. The 2 × 2 ANOVAs of inactive lever responses during the final post-MR EXT vs Test session did not reveal significant *Context* × *Treatment* interaction, *Context* or *Treatment* main effects.

The 2 × 6 ANOVA of lever responses during 20 min Test Bins of the 2 h Test revealed significant *Test Bin* main effect (active: F_5, 14_ = 25.197, p < 0.001; inactive: F_5, 14_ = 8.955, ^#^p < 0.001) with no *Test Bin* × *Treatment or Treatment* main effects (Fig. 3E). Tukey’s post-hoc comparisons revealed that responses for both VEH and CHX groups decreased by the final 20 min of the Test (active and inactive: Bin 1 > 6, ^#^p < 0.01). Further analysis of lever responses during 5 min Bins of the 2 h Test did not reveal *Bin* × *Treatment* interaction, *Bin* or *Treatment* main effects at the active or inactive levers (Suppl Fig. 3B).

### Adolescent behavior during Coc-SA, EXT, MR and tests: effect of CHX on reconsolidation of cocaine context memories

Adolescent rats in the VEH (n = 6) and CHX (n = 8) groups displayed similar responses on both levers with no pre-existing differences observed in the number of sessions to acquire Coc-SA, lever responses, or cocaine infusions during the last three sessions of Coc-SA (Fig. 4A). The 2 × 10 ANOVAs during Coc-SA did not reveal significant *Coc-SA session* × *Treatment* or *Coc-SA infusion* × *Treatment* interaction effects.

Adolescent rats in the VEH and CHX groups had similar lever responses during EXT training (Fig. 4B). The 2 × 8 ANOVA of lever responses revealed a significant *EXT session* main effect (active: F_7, 12_ = 19.713, p < 0.001; inactive: F_7, 12_ = 14.304, p < 0.001), with no *EXT session* × *Treatment* interaction or *Treatment* main effects. Tukey’s post-hoc comparisons revealed that responding decreased in both VEH and CHX groups by the final EXT session (active and inactive: EXT S1 > S8, ^#^p < 0.01).

Adolescent rats in the VEH and CHX groups had similar lever responses during the MR session (Fig. 4C). The 2 × 3 ANOVA of lever responses during 5 min bins of the 15 min MR session revealed significant *MR Bin* main effect (active: F_2, 12_ = 8.311, p = 0.002) with no *MR Bin* × *Treatment* interaction or *Treatment* main effects. Tukey’s post-hoc comparisons revealed that only VEH groups displayed reduced active lever responses by the final 5 min of MR (VEH: Bin 1 > 3, ^#^p < 0.01; CHX: Bin 1 ≠ 3, 2 ≠ 3). Tukey’s post-hoc comparisons did not reveal significant differences in inactive lever responses during 5 min MR Bins (Suppl Fig. 4A).

Adolescent rats in the VEH and CHX groups displayed increased cocaine-seeking behavior when exposed to the cocaine-paired context (Fig. 4D). The 2 × 2 ANOVA of active lever responses during the final post-MR EXT vs Test sessions revealed a significant *Context* main (active: F_1, 12_ = 31.633, p < 0.001; inactive: F_1, 12_ = 7.010, p = 0.021) with no *Context* × *Treatment* interaction or *Treatment* main effects. Tukey’s post-hoc comparisons revealed that lever responses in the VEH and CHX groups were higher during the Test compared to the final post-MR EXT session (active: ^#^p < 0.01; inactive: ^#^p < 0.05) therefore, both VEH and CHX-treated adolescent groups exhibited cocaine-seeking behavior.

The 2 × 6 ANOVA of lever responses during 20 min Test Bins of the 2 h Test revealed significant *Test Bin* main effect (active: F_5, 12_ = 18.524, p < 0.001; inactive: F_5, 12_ = 6.149, p < 0.001) with no *Test Bin* × *Treatment or Treatment* main effects (Fig. 4E). Tukey’s post-hoc comparisons revealed that active lever responses for both VEH and CHX groups, and inactive for the CHX group decreased by the final 20 min of the Test (Bin 1 > 6, active: ^#^p < 0.01; inactive: ^#^p < 0.05). Further analysis of lever responses during 5 min Bins of the 2 h Test revealed *Bin* main effect (active: F_2, 12_ = 8.618, p = 0.002; inactive: F_2, 12_ = 4.441, p = 0.023), and *Bin* × *Treatment* interaction for active lever (F_2, 24_ = 3.972, p = 0.032); however Tukey’s post-hoc comparisons revealed no significant effects at Test Bins within or between groups (Suppl Fig. 4B).

## Discussion

Overall, systemic protein synthesis inhibition after retrieval of cocaine-associated contextual memories did not reduce relapse-like behavior in adult or adolescent cocaine-exposed groups. The current results did not reveal age-dependent differences during operant phases of behavioral training (Fig. 2). Adult and adolescent rats had similar lever responses during Coc-SA and EXT training phases and these data are in line with previous reports in which self-administration of cocaine occurred in the presence of diffuse environmental stimuli or paired with explicit cues^27–29,35,36^.

There is evidence that adolescent rats have increased resistance to extinguish fear and cocaine-related behaviors^24,29,37^. A previous study determined that adolescent rats were less responsive to a rodent version of cue-exposure therapy (CET). In this study, investigators combined a Coc-SA paradigm with passive presentation of a cocaine-associated cue to mimic human CET and observed that adult and adolescent rats displayed similar levels of EXT training behavior, as observed in our study, but were resistant to passive cue extinction compared to adults^29^. We have also noted a potential resistance to extinguish lever responses in adolescent rats within the original cocaine-paired context, but not within the EXT context^28^. Similar to our previous reports, adolescent rats had higher total inactive lever presses during reinstatement tests; however both age groups displayed increased inactive presses during the first 20 min bin of the reinstatement test. It is possible that across the entire test session, adolescent cocaine-exposed rats had increased motivation or response generalization to both levers in the drug-paired context^38,39^.

We modified our ABRV Coc-SA paradigm to incorporate standard procedures to test whether protein synthesis was required for the reconsolidation of adult- and adolescent-formed cocaine-context memories. Importantly, lever responses were similar between adult and adolescent cocaine-exposed rats during a reactivation session used to retrieve cocaine-context memories. We chose 15 min of exposure to the previous cocaine-paired context since this timeframe is sufficient to elicit retrieval in adult rats without engaging extensive extinction processes^11,12,40^. We examined 5 min intervals of the 15 min MR session but did not observe age-dependent differences in the curves of lever responses (Suppl Fig. 2A) or in the latency for the first lever press during MR; therefore, the MR session likely served to retrieve cocaine-associated memories to a similar extent for both age groups. Overall, operant lever response behavior was similar between adult and adolescent rats during Coc-SA and EXT training and MR, which suggests that an age-dependent difference in the strength of drug-context associations did not contribute to the lack of effect of CHX on reconsolidation and subsequent relapse.

The current results revealed that adult and adolescent cocaine-exposed rats had similar magnitudes of context-induced cocaine-seeking behavior regardless of post-retrieval treatment with VEH or CHX (Figs. 3, 4). Surprisingly, CHX treatment failed to reduce cocaine-seeking behavior in adult rats which contrasts with reports of attenuated cocaine reward and cue-induced reinstatement following systemic CHX administration^21,41^. A subcutaneous dose of 1.0 mg/kg CHX has been shown to reduce protein synthesis in the brain by up to 57%^42^. A range of CHX doses, from 1.0 to 15 mg/kg, has been used for cocaine CPP and operant self-administration studies. A systemic dose of 2.2 mg/kg CHX reduced cue-induced cocaine-seeking behavior; whereas a 1.0 mg/kg dose did not reduce seeking behavior^21^. Higher doses of systemic CHX (15 mg/kg) resulted in reduced cocaine CPP^41,43^. We therefore chose the lowest dose of CHX that was within the range expected to observe behavioral effects, to avoid potential negative effects related to toxicity with protein synthesis inhibitors in general^44,45^. Weight loss has been noted after SC administration of CHX at a dose of 1.5 mg/kg; therefore, we examined weight at similar intervals—2 days before and after VEH or CHX administration^45^. Adult and adolescent groups both gained weight at similar magnitudes, with no reductions in weight observed in CHX-treated groups (Suppl Fig. 1). The most parsimonious explanation would suggest unsuccessful CHX delivery, however differences in experimental procedures and food delivery could also account for these results. Rats in the current study received restricted, rather than ad libitum feed. Both age groups gained weight on restricted feed, but in combination with a history of cocaine intake, our experimental procedures may have obscured the ability to detect subtle differences in weight loss due to CHX administration.

The current study used comparable lengths of memory reactivation and doses of CHX, but a major difference from previous reports was the type of stimuli used to elicit retrieval. Previous studies that reported CHX-induced impairment in reconsolidation and drug-seeking behavior used a cocaine-associated explicit cue to elicit retrieval, while the current study exposed rats to a previous cocaine-paired context. It is known that different brain regions and circuits support cue vs contextual cocaine-seeking behavior^7,32,46^. Furthermore, signaling cascades that are important for memory reconsolidation are shown to be differentially involved in cue vs contextual retrieval and subsequent relapse behavior. For example, post-MR inhibition of protein kinase A activity in the BLA impairs both cue- and context-elicited reconsolidation and subsequent cocaine-seeking; whereas post-MR inhibition of CAMKII in the BLA only impairs cue-elicited drug-seeking behavior^12,18,40^. Another difference from previous studies is that reinstatement Tests in the current study were conducted 2 days after MR and CHX treatment, whereas previous reports conducted Tests 3 days after MR + CHX treatment. The lack of a behavioral effect on relapse from adult systemic CHX manipulation likely is specific to retrieval of contextual vs cue memories, as has also been observed in the fear conditioning literature^47^.

While beyond the scope of the current work, future studies could focus on use of the unconditioned stimuli (US) of cocaine to retrieve and destabilize cocaine-associated memories. Previous reports that used the US to retrieve and destabilize both drug CPP and operant drug-associated memories observed reduced reward and cue-induced cocaine-seeking in adult rats^48–51^. Use of the US to retrieve cocaine-linked memories could be a particularly relevant approach in adolescent rats, given that this age group displays higher stress and cocaine-primed reinstatement compared to their adult counterparts, suggesting potential age differences in reactivity to external vs interoceptive cues^33,52^. In line with modifying the stimuli used to retrieve cocaine-context memories, future studies could present a CS-US pairing that differs in duration from the original CS-US used during training to provide for a larger prediction error during the memory retrieval session^53^.

Lastly, it is also possible that systemic administration of CHX may have exerted effects in several brain regions involved in reconsolidation and motivated behavior, thereby obscuring potential reductions in cocaine-seeking behavior. A large body of literature has demonstrated brain-specific reconsolidation mechanisms contribute to relapse-like behavior^11,12,20,34,54^. Delivery of protein synthesis inhibitors to specific brain regions to investigate adult vs adolescent differences in drug memory reconsolidation may be a more promising avenue for future experiments.

## Conclusions and future directions

The current results suggest that both adult and adolescent-formed cocaine-context associations were not sensitive to, or weakened by, reconsolidation manipulations via systemic protein synthesis inhibition. A large body of literature demonstrates that brain-specific reconsolidation mechanisms contribute to cocaine-associated memories and relapse. Therefore, future directions should investigate the brain region specific mechanisms that strengthen cocaine-context memories in adult and adolescent rats. We recently demonstrated that adolescent cocaine-exposed rats display a time-dependent increase in context-induced cocaine-seeking behavior after 15 days of abstinence from cocaine intake, whereas adult cocaine-exposed counterparts do not^28^. Future studies could aim to use similar procedures of memory reactivation in a cocaine-paired context after extinction training and then examine whether reduced context-induced incubation of craving is observed in adolescent cocaine-exposed rats.

### Supplementary Information

Supplementary Legends.

Supplementary Figure 1.

Supplementary Figures.

## Data Availability

The datasets used and/or analyzed during the current study available from the corresponding author on reasonable request.
