# Supplementary material for: Influence of reconsolidation in maintenance of cocaine-associated contextual memories formed during adolescence or adulthood
Source: Sci Rep. 2023 Aug 25;13:13936. doi: 10.1038/s41598-023-39949-y (PMC10457301; doi:10.1038/s41598-023-39949-y)
Supplement: Supplementary file 1 — Supplementary Legends. [file 41598_2023_39949_MOESM1_ESM.docx]

Original Investigation to *Scientific Reports, Models of Addiction Collection*

*Supplemental Figure Legends*

**Influence of reconsolidation in maintenance of cocaine-associated contextual memories formed during adolescence or adulthood.**

Andre N. Herrera Charpentier, Doris I. Olekanma, Christian T. Valade, Christopher A. Reeves, Bo Ram Cho, Amy A. Arguello^*^

Psychology Dept., Michigan State University,

Interdisciplinary Science and Technology Building, West Rm. 4010

766 Service Rd., East Lansing, MI, 48824, USA

*Corresponding Author:

Dr. Amy A. Arguello

Michigan State University (MSU)

Department of Psychology, Behavioral Neuroscience

Interdisciplinary Science and Technology Building, West Rm. 4010

766 Service Rd

East Lansing, MI 48824

Phone: 214-912-8911

Email: [arguell5@msu.edu](mailto:arguell5@msu.edu)

**Supplementary FIGURE LEGENDS**

**Supplementary Figure 1: Effect of CHX treatment on adult and adolescent weight gain.**

Mean ± SEM of weight gain (gm) for adult and adolescent rats 2 days prior and 2 days after vehicle or cycloheximide (VEH, CHX) administration. Rats received subcutaneous injection of VEH or 2.5 mg/kg CHX immediately after a 15min memory reactivation (MR) session. No significant *Weight x Treatment* interaction or main effects were observed for adult or adolescent groups. Adult groups denoted by circles: Blue = adult VEH (n=8), Black = adult CHX (n=8). Adolescent groups denoted by squares: Orange = adol VEH (n=6), Black = adol CHX (n=8).

**Supplementary Figure 2: Effect of age on memory reactivation**

Mean ± SEM of active and inactive lever responses for adult and adolescent rats during **(A)** 5min Bins of the 15min memory reactivation session (**MR)** in the previous cocaine-paired context, before rats received subcutaneous injection of vehicle or 2.5 mg/kg cycloheximide (VEH, CHX). Symbols indicate significant within-subject differences revealed by Tukey’s test

**(A)** ^#^ p<0.05: active lever 5min MR Bins 1>3, for adult and adolescent groups. Groups denoted by: Blue = adult (n=16), Orange = adolescent (n=14).

**Supplementary Figure 3: Memory reactivation and effect of cycloheximide on reconsolidation of cocaine-context memories in adult cocaine-exposed rats.**

Mean ± SEM of active and inactive lever responses for adult rats during **(A)** 5min Bins of the 15min memory reactivation session (**MR)** in the previous cocaine-paired context, before rats received treatment and during **(B)** 5min Bins of the reinstatement test (Test) after rats received subcutaneous injection of vehicle or 2.5 mg/kg cycloheximide (VEH, CHX). Symbols indicate significant within-subject differences revealed by Tukey’s test **(A)** ^#^ p<0.01: active lever 5min MR Bins 1>3, for VEH and CHX groups. Groups denoted by: Light blue = adult VEH (n=8), Black = adult CHX (n=8).

**Supplementary Figure 4: Memory reactivation and effect of cycloheximide on reconsolidation of cocaine-context memories in adolescent cocaine-exposed rats.**

Mean ± SEM of active and inactive lever responses for adolescent rats during **(A)** 5min Bins of the 15min memory reactivation session (**MR)** in the previous cocaine-paired context, before rats received treatment and during **(B)** 5min Bins of the reinstatement test (Test) after rats received subcutaneous injection of vehicle or 2.5 mg/kg cycloheximide (VEH, CHX). Symbols indicate significant within-subject differences revealed by Tukey’s test

**(A)** ^#^ p<0.01: active lever 5min MR Bins 1>3, for VEH group. Groups denoted by: Light orange = adolescent VEH (n=6), Black = adolescent CHX (n=8).
